# Distinct Transcript Isoforms of the Atypical Chemokine Receptor 1 (*ACKR1*) / Duffy Antigen Receptor for Chemokines (*DARC*) Gene Are Expressed in Lymphoblasts and Altered Isoform Levels Are Associated with Genetic Ancestry and the *Duffy-Null* Allele

**DOI:** 10.1371/journal.pone.0140098

**Published:** 2015-10-16

**Authors:** Melissa B. Davis, Andrea Walens, Rupali Hire, Kauthar Mumin, Andrea M. Brown, DeJuana Ford, Elizabeth W. Howerth, Michele Monteil

**Affiliations:** 1 Department of Genetics, Franklin College of Arts and Sciences, University of Georgia, Athens, GA, United States of America; 2 Department of Pathology, College of Veterinary Medicine, University of Georgia, Athens, GA, United States of America; 3 Department of Molecular Biology and Biochemistry, Georgia Regents University–University of Georgia Medical Partnership, Athens, GA, United States of America; Rutgers University, UNITED STATES

## Abstract

The Atypical ChemoKine Receptor 1 (*ACKR1*) gene, better known as *Duffy* Antigen Receptor for Chemokines (*DARC* or *Duffy*), is responsible for the *Duffy* Blood Group and plays a major role in regulating the circulating homeostatic levels of pro-inflammatory chemokines. Previous studies have shown that one common variant, the *Duffy Null (Fy-)* allele that is specific to African Ancestry groups, completely removes expression of the gene on erythrocytes; however, these individuals retain endothelial expression. Additional alleles are associated with a myriad of clinical outcomes related to immune responses and inflammation. In addition to allele variants, there are two distinct transcript isoforms of *DARC* which are expressed from separate promoters, and very little is known about the distinct transcriptional regulation or the distinct functionality of these protein isoforms. Our objective was to determine if the African specific *Fy-* allele alters the expression pattern of *DARC* isoforms and therefore could potentially result in a unique signature of the gene products, commonly referred to as antigens. Our work is the first to establish that there is expression of *DARC* on lymphoblasts. Our data indicates that people of African ancestry have distinct relative levels of *DARC* isoforms expressed in these cells. We conclude that the expression of both isoforms in combination with alternate alleles yields multiple Duffy antigens in ancestry groups, depending upon the haplotypes across the gene. Importantly, we hypothesize that *DARC* isoform expression patterns will translate into ancestry-specific inflammatory responses that are correlated with the axis of pro-inflammatory chemokine levels and distinct isoform-specific interactions with these chemokines. Ultimately, this work will increase knowledge of biological mechanisms underlying disparate clinical outcomes of inflammatory-related diseases among ethnic and geographic ancestry groups.

## Introduction

The *Duffy Antigen Receptor for Chemokines (DARC)*, recently renamed *Atypical Chemokine Receptor 1 (ACKR1)*, expresses the red blood cell antigens that define the *Duffy* Blood Groups for which it was originally discovered [1, 2]. Much of the research involving *DARC/ACKR1* deals with its role as the receptor for the malarial parasites *Plasmodium vivax* and *Plasmodium knowlesi* [3–5]. It is now known to be a promiscuous atypical chemokine receptor [6], interacting with an array of both classes of chemokines (C-C-L and C-X-C-L); including those involved in inflammation and angiogenesis [3, 7, 8]. *DARC/ACKR1* is ubiquitously expressed and highly conserved across placental mammalian species (*Boreoeutheria*) with over 90% amino acid conservation across primates and over 70% nucleic acid conservation in lower mammals[9]. The main normal function described for DARC/ACKR1 is that it effectively sustains homeostatic levels of circulating chemokines and modulates chemokine gradients between tissues and blood to mediate the influx of neutrophils and monocytes from blood vessels into tissues [10, 11] during immune responses. *DARC/ACKR1* has also been implicated to affect cancers as a pro-inflammatory cytokine receptor, specifically in lung cancer etiology, BrCa progression by *in vitro* studies and allele-specific BrCa patient survival [12–14]. While these studies implicate *DARC/ACKR1* in cancer processes, there are still lingering questions concerning how *DARC/ACKR1* lends its chemokine binding capacity toward cancer progression. In fact, there is very little investigation of DARC/ACKR1 in regard to the complexity of gene product variants and their distinct role in biological outcomes of immune/inflammatory responses during tumorigenesis.

**Fig 1:**
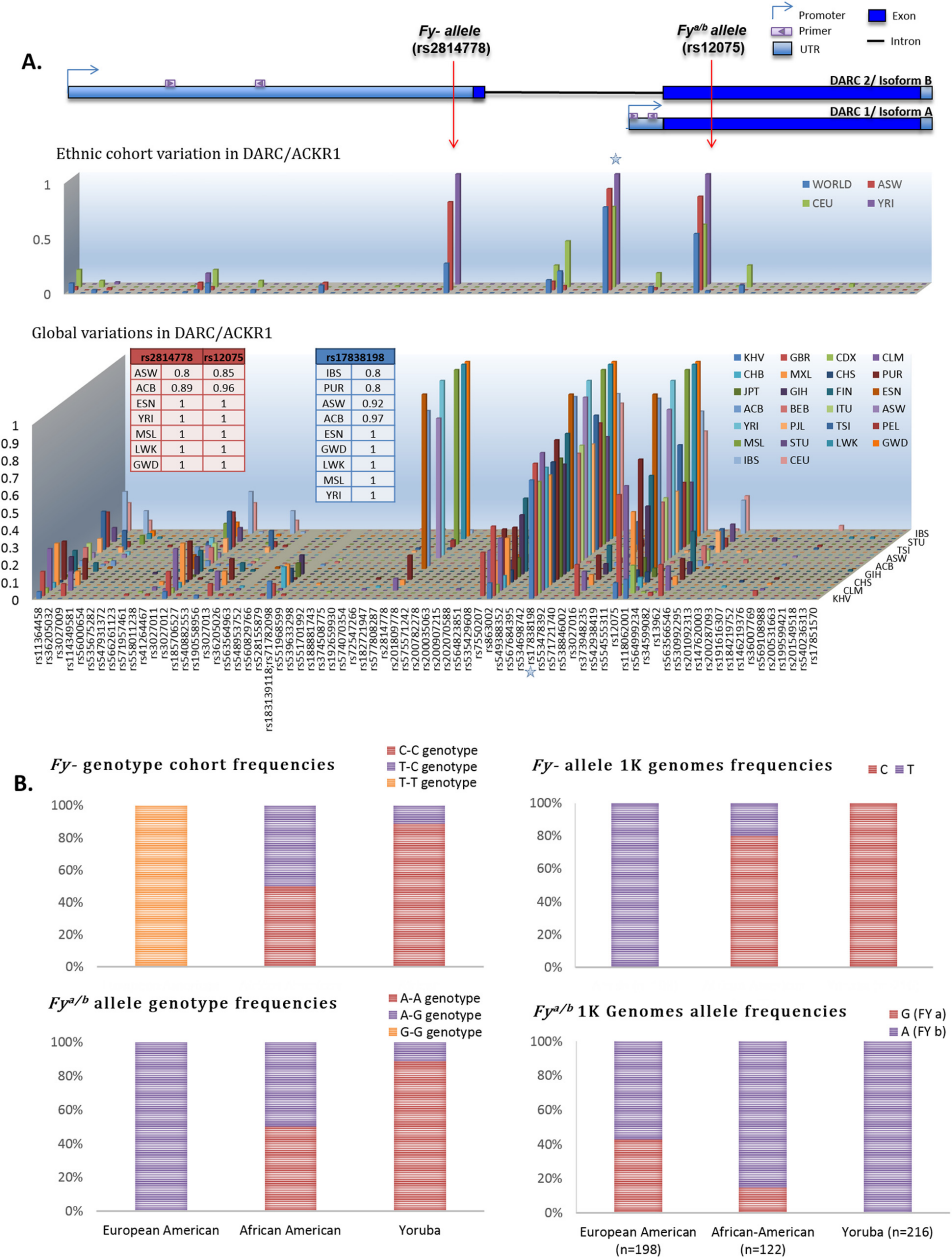
The DARC gene structure and frequencies of the two major alleles; *Fy-* and *Fy* ^*a/b*^ in HAPMAP experimental cohort compared to entire 1000 Genomes (1K) populations. **(A).** Top, Model of DARC/ACKR1 gene structure indicating the two gene promoter-driven isoforms, DARC1/A and DARC2/B. Primers used for qPCR of transcript variants are indicated as double arrows. Also indicated are the *Duffy Null* allele (rs2814778) and *Fy* ^*a/b*^ allele (rs12075). The two transcript products result in unique gene products; Duffy isoform A has 338aa and Duffy isoform B has 336aa. Top graph shows the allele frequencies of all documented polymorphisms in the DARC gene region, specifically in the ethnic cohorts of interest; African American (ASW), West African- Yoruba in Ibadan (YRI) and European American (CEPH). The overall ‘world’ variant frequencies are also indicated, summarizing all sampled populations in the 1000 Genomes. The bottom graph details the individual ancestry groups that encompass the global frequencies across the entire 1000 Genomes dataset for the entire DARC gene region. The X-axis matches the gene model with regard to relative gene structure location. The rs# IDs on the X axis of the bottom graph correspond to those on the top graph, for each SNP. The starred locus indicates a deletion polymorphism that resides in the promoter region of the DARC1/A isoform that has not previously been explicitly reported, described in the text (*rs17838198)*. This locus appears to have been under some selection influence in other populations, similar to the as the malaria selection influence on the *Duffy Null* allele in African populations, as it is the 3rd most prominent variant in the gene and prevalent in most populations. **(B).** The genotype frequencies for the *Fy-* and *Fy* ^*a/b*^ loci in our cohort are shown in the inset panel on the left. The allele frequencies of *Fy-* and *Fy* ^*a/b*^ for the entire corresponding ancestry 1K Genomes panels are shown in the inset panel on the right. The genotype percent distributions of the *Fy-* genotype across our West African—YRI, African American and European American subpopulations correlate with the high prevalence of the alleles in 1,000 Genome populations. Nearly half of the AA/ASW group is heterozygotes with an additional 40% being CC homozygotes, indicating more than 90% have the *Fy-* allele, as seen in the adjacent graph. The allele distribution table shows proportion values correlate with previously established numbers and validate our population.

The *DARC/ACKR1* gene products are traditionally referred to as “*Duffy* blood group antigens” and vary in their expression among different human populations in several ways. There are several antigens described in various clinical reports which presumably represent variants of the gene product [15]. However, the most commonly described variants are those derived from the two major ***DARC*** ***alleles*** resulting from common Single Nucleotide Polymorphisms (SNPs). One SNP occurs in the gene regulatory region and yields the “**Fy- allele**” or “***Duffy Null***” (*rs2814778* ***)***, and the other SNP is a missense mutation that yields the “***Fy B***” and “***Fy A***” alleles (rs12075). The *Duffy Null* allele nomenclature refers to a phenotype also known as “Erythrocyte Silent” (*Fy* ^*es*^) removing expression of *DARC* on red blood cells (RBCs) and is usually denoted as “Fy^-a-b^” to reflect the missing blood group antigens. This allele is rare among individuals of either European or Asian descent but is the most common phenotype in most Africans and African Americans. The *Fy*- allele has a frequency of nearly 100% in West Africans and greater than 80% of African Americans (Fig 1) [16, 17] as it confers resistance to malaria [18] and became fixed in African populations where malaria is endemic[4, 17]. In addition to these *DARC* variants, there are 4 other alternate *Duffy* blood group antigens, usually interpreted as *DARC* gene allele variants, namely; *Fy*3, *Fy*4, *Fy*5 and *Fy*6. However, the specific immunogenic domain that defines most of these antigens is not fully elucidated [15, 19]. Of note, one additional important yet rare phenotype, *Fy* ^x^, expresses the *Fy* ^b^ allele, but is believed to have ‘weak’ expression that is not always detected by the anti-Fy^b^ antibody [6, 20], suggesting the epitope is unique and possibly a yet undescribed additional Duffy antigen. One potential explanation for such complexity among Fy antigens could be the co-expression of alternative *DARC* gene product isoforms and distinct post-translational modifications (i.e. glycosylation) between the isoforms acting as immunogens. This possibility has not yet been addressed.

There is long-standing evidence of two separate ***DARC*** ***isoforms*** *[21]* that are derived from separate promoters and yield distinct protein products. Specifically, *DARC* yields two isoform transcripts [17] *DARC*1/Isoform A (NM001122951.2/UniProt Q16570-2) and *DARC*2/Isoform B (NM00236.3/UniProt Q16570-1) [22] that create distinct gene products. Each isoform has unique N-terminal amino acids in the extracellular topological domain where chemokine binding occurs. The isoforms contain 17 unique amino acids between them that include distinctive glycosylation and enzymatic cleavage sites. Therefore, there is great potential for the isoforms to diverge in their functionality and lend a level of dynamic complexity to the role of *DARC* in chemokine regulation. To date, no study has addressed the potential differences in functionality between the isoforms. However, there is clinical evidence of complexities in the *Duffy* blood group beyond what has been previously described. Specifically, we observe complexity beyond the two major DARC alleles in presentations of varied clinical manifestations linked to *DARC* including, neutropenia [23, 24], organ system damage in sickle cell disease[25], cancer metastasis regulation[26] as well as hemolytic complications arising from immunological responses among *Duffy* antigens during blood transfusions [27]. At least in part, these observations could be explained by considering the alternative protein isoforms which would increase the repertoire of antigens 2-fold. In this work, we have shown that both of the *DARC* isoforms are expressed in lymphoblast lines and at varying levels, relative to the African-specific *Fy*- allele genotype.

## Materials and Methods

### Cohort design and 1000 Genomes data

Our study population is a well-defined subset of diverse genetic ancestry groups from genetically described individuals of the International Human Genome Haplotype Mapping Project (HAPMAP)[28–32]. We obtained a subset of HAPMAP lymphoblast cell lines to create a representative panel of genetic ancestry groups, including: Yoruba in Ibadan, Nigeria (abbreviation: YRI); Japanese in Tokyo, Japan (abbreviation: JPT); Han Chinese in Beijing, China (abbreviation: CHB); CEPH (Utah residents with ancestry from northern and western Europe) (abbreviation: CEU). Each of our investigations utilized at least 20 cell lines across the ancestry categories. We obtained access to the 1000 Genomes[33] dataset through the public access portal at www.1000genomes.org.

### Genotyping and allele distributions

We conducted independent validation of *DARC*/*ACKR1* allele genotypes in our cohort samples. *Duffy Null* genotyping was done with PCR amplification and Sanger sequencing. Primers were designed to flank the *Fy*-. Allele and genotype distributions were then assessed for representative population accuracy in each ancestry group, using GenePop version 4.2 Option 5, to characterize allele frequencies and Fis[34]. *Fy* ^*a/b*^ allele genotyping was done with the TaqMan Allelic Discrimination protocol. To assess the ancestry distribution in comparison to our results, we utilized the full population genotype set, conducting allele distribution calculations for all SNPs reported in the gene region. We extracted the distributions for the two alleles of interest and they are displayed in Fig 1.

### Gene Expression

For *in vitro DARC/ACKR1* gene expression evaluations, we used comparative quantitation methods to determine the relative mRNA levels of *DARC/ACKR1* isoforms among cell lineages derived from 3 distinct ancestry groups. Specifically, after detection of the transcript with Reverse Transcription PCR, we conducted Real-time PCR using SABiosciences SYBR green protocol on Applied Biosystems 7500 cycler and SDS software version 1.4. The Ct values were transformed to dCT values using RPL13 endogenous control gene, displayed as 1/dCT to preserve directional interpretation of expression levels. We used the manufacturer’s suggested protocols for all assays and primers. Isoform specific primers were designed using the primer-BLAST program through NCBI [35]. Primer sequences were as follows:

*DARC*1 expression Forward-TCTGGGTATGTCCTCCAGGC

*DARC*1 expression Reverse-AAGGGCAGTGCAGAGTCATC

*DARC*2 expression Forward-TCCAATTTCCCAGCACCTCC

*DARC*2 expression Reverse-GGCTGGTTGGGACTACACTC

For *in situ DARC/ACKR1* gene product evaluations in tissues and cells, we used immunohistochemistry to assess the *DARC/ACKR1* protein expression. Formalin-fixed cultures were paraffin embedded and sections stained for *DARC*/*ACKR1* using a goat anti-*DARC* antibody (Novus biologicals: NB100-2421) and detection with biotinylated anti-goat (Biocare), alkaline phosphatase streptavidin (Biogenex), and fast-red chromogen (Biocare).

## Results and Discussion

### Global and cohort distributions of DARC variants

The global distributions of *Duffy Null (Fy*-) genotypes have been previously reported in several studies [36–39]. In relation to the gene’s structure, the *Fy*- SNP involves a T to C substitution in the promoter region of the *DARC2* isoform and in the 5’ Untranslated Region (UTR) of the *DARC1* isoform (Fig 1A). In our study cohort, this *Fy*- phenotype is present in over 98% of West African Nigerians in Idaban (YRI) and in 50–80% of African Americans (AA/ASW)[16] and less than 1% of European Americans (CEPH). Intriguingly, we have revealed a previously unexplained variant (rs17838198) that appears to be in Linkage Disequilibrium (LD) with both the *Fy-* allele and the *Fy* ^*a/b*^ allele, according to the HapMap data for our ancestry groups of interest. Upon expanding our investigation to include all ancestry groups of the 1,000 Genomes dataset [33] (Fig 1A, S1 Table), we see that *rs17838198* is even more prevalent than the *Fy* ^*a/b*^ allele across most ancestry groups and just as conserved in African populations as the *Fy-* allele, according to the Minor Allele Frequency data generated by the 1,000 Genomes Project[33]. Of note, *rs17838198* is a single nucleotide insertion/deletion adjacent to the DARC1 isoform promoter with unknown effect that could potentially alter DARC gene expression if a transcription factor or polymerase complex binding site is disrupted. We will investigate this possibility further in a later publication.

For this investigation of DARC isoform regulation, we conducted independent genotyping of our HAPMAP [32] sub-population panel to ensure our cohort reflects the expected distributions for each ancestry group. Both the *Fy-* allele and the *Fy* ^*a/b*^ allele genotype distributions show the expected pattern of association with ancestry (Fig 1B). All CEPH individuals are TT homozygotes (*Fy+*), the West Africans-YRI are primarily CC homozygotes (Fy-) with less than 30% being heterozygotes and no TT homozygotes. The African-American group has the highest percentage of heterozygotes and could potentially be uniquely affected by the combination of alleles. The complete lack of TT homozygotes in the African population, with only a single individual with heterozygote status in the entire 1,000 genomes sample set, reaffirms the extent of which natural selection has occurred for the C allele in African regions. The Fy^a/b^ locus also shows allele association with ancestry, with the majority of YRI having the A allele, which would confer expression of the *Fy* ^*b*^ antigen in these individuals. The CEPH population has the highest prevalence of the B allele though all are heterozygotes state. This will result in expression of both the *Fy* ^*a*^ and *Fy* ^*b*^ antigen on erythrocytes (*Fy* ^*a+b+*^) in this population, as well as on lymphoblasts that we will discuss later. The genotype frequency in AA/ASW individuals appears to be an even mix of both the YRI and CEPH, which is expected given the AA/ASW group is an admixed population, largely made up of both West African and European-American ancestry. Hence, our correlative findings between the 1,000 Genomes and our cohort indicate our representative cohort does reflect the global population of each ancestry group. Interestingly, the YRI individual who is heterozygous for the *Duffy Null* allele has two copies of the *Fy* ^*b*^ allele, consistent with prior studies indicating that the Fy(a-b+) phenotype is most prevalent among Blacks who are not *Duffy Null*, meaning the *Fy* ^*b*^ allele is segregating with the *Duffy Null* allele in this population. We can see this most pointedly demonstrated the AA/ASW group, which has the highest percentage (50%) of heterozygotes for the *Duffy Null* allele. In fact, the AA/ASW population has compound heterozygotes for the *Fy-* allele and the *Fy* ^*a/b*^ allele which should have a unique effect on expression of antigens.

### *DARC* protein product is expressed in lymphoblasts and shows dynamic expression levels across *Fy*- and ancestry groups

**Fig 2:**
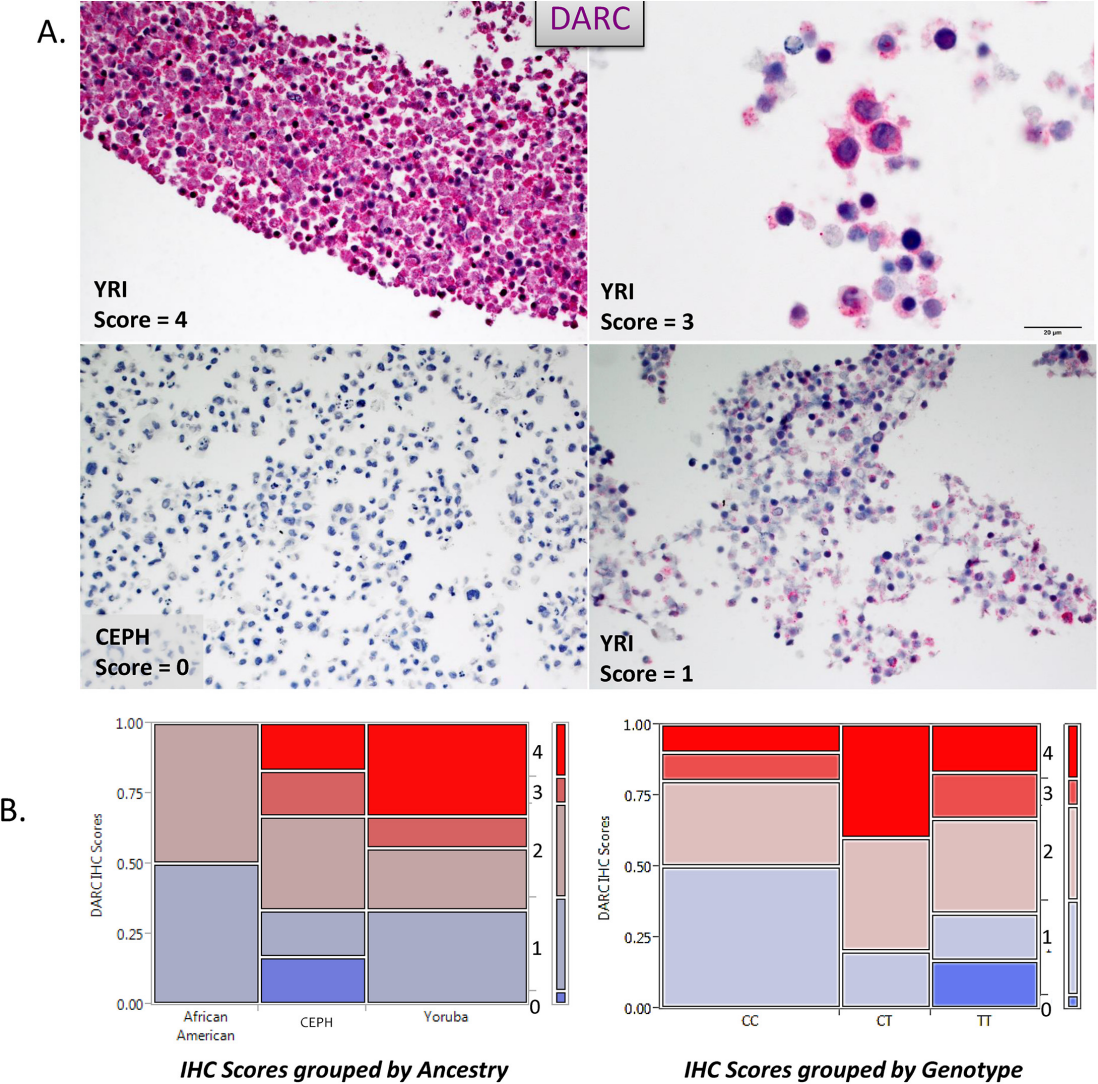
DARC/ACKR1 protein is differentially expressed among lymphoblasts derived from divergent ancestry groups in HAPMAP/ 1,000 (1K) Genomes populations. **(A).** Representative IHC images of DARC expression in the lymphoblasts of Africans (YRI) and European Americans (CEPH/CEU). DARC is stained with Vulcan Red chromogen nuclei are stained blue with hematoxylin. **(B).** Shows a distribution analysis of IHC scores across ancestry groups and *Fy-* genotypes. The full range of scores (0–4) were observed in our cohort, with clear trends within groups. European Americans were the only ancestry group to have a 0 score, indicating no DARC expression in these cells, correlating with lower transcription of the gene. Similarly, individuals with a homozygous *Duffy-positive* (TT) genotype were the only to have a score of 0, indicating that lowest levels of DARC expression in lymphoblast are associated with the European lineage and the TT genotype, in contrast to higher DARC expression in the lymphocytes of African lineages. Accordingly, the majority of high lymphoblast IHC scores (3 or 4) were in the African lineage. All African Americans only showed moderate expression levels in lymphoblasts.

In order to investigate ancestry related DARC distinctions, we chose to utilize the lymphoblast lines from the HAPMAP resources. Several genome-wide investigations have indicated DARC/ACKR1 expression in many tissues [40–45], including low level transcripts from microarray data in the cell lineages that were used to derive the HAPMAP lines (S1 Fig). However, there were no explicit reports of these cells expressing the DARC/ACKR1 gene product. Therefore, we conducted IHC staining of our set of HAPMAP lines to measure DARC/ACKR1 protein expression. We observed a large amount of variation of expression across the cohort, documenting the full spectrum of IHC scoring; from 0 (no expression) to 4 (very high expression) (Fig 2A). An analysis of the IHC score distributions among ancestry groups and ***Fy-*** genotypes suggests a trend of expression that may be linked to ancestry (Fig 2B). We observed that only the CEPH/CEU ancestry group and the homozygous TT (***Fy+***) genotype groups have individuals who displayed a score of 0, indicating no expression. Over half of this group only had low to moderate expression (score of 1–2) which correlates with our observed lower transcript levels of the DARC isoforms as well. While this might indicate that some individuals of European descent may not typically express high levels of DARC/ACKR1 in lymphoblasts, almost half of this group also displayed high levels of DARC/ACKR1. This suggests that DARC/ACKR1 expression is highly variable in these cells. In contrast, we observe that the YRI and ASW groups primarily displayed the highest scores (3) indicating very high levels of DARC/ACKR1 product in lymphoblasts from these groups. This suggests that these groups would typically have higher levels of DARC/ACKR1 expression in these cells, with few individuals showing low levels.

All in all, published reports and data sources that indicate little to no expression in these cells are likely biased with over-representation of samples and data from people of European ancestry, with very few samples from people of African ancestry. Such bias leads to incorrect conclusions concerning the spatial expression pattern of DARC/ACKR1 (S1 Fig), and specifically now in lymphoblasts. While our small sample size would not avail statistical significance in differences among the CEPH/CEU, YRI and AA/ASW groups, we have clearly demonstrated that DARC/ACKR1 is expressed in these cells, at varying levels among individuals; including, extremely high protein product levels among the CEPH and YRI individuals (Fig 2A). These findings indicate that our HAPMAP lines are ideal for investigations of differential DARC expression and regulation.

### Both *DARC1 and DARC2* isoforms are expressed in lymphoblasts and have distinct expression levels

We next investigated whether the DARC protein products expressed by lymphoblasts could be the result of distinct DARC isoform expression. Because the ancestry-specific *Fy-* allele results in a cell-type-specific loss of *DARC* expression, it was plausible that there is dynamic regulation of the gene in certain contexts, given that individuals who lack erythrocyte DARC/ACKR1 expression still express the gene in other tissues, such as endothelial tissues [7, 46–48]. In addition, previous reports indicated that the erythrocyte silencing of *DARC/ACKR1* results from loss of a single transcription factor binding site. Therefore, given the isoforms are expressed from distinct promoters, we hypothesized that there may be significant variation of *isoform expression* throughout the global population associated with the *Fy*- allele.

**Fig 3:**
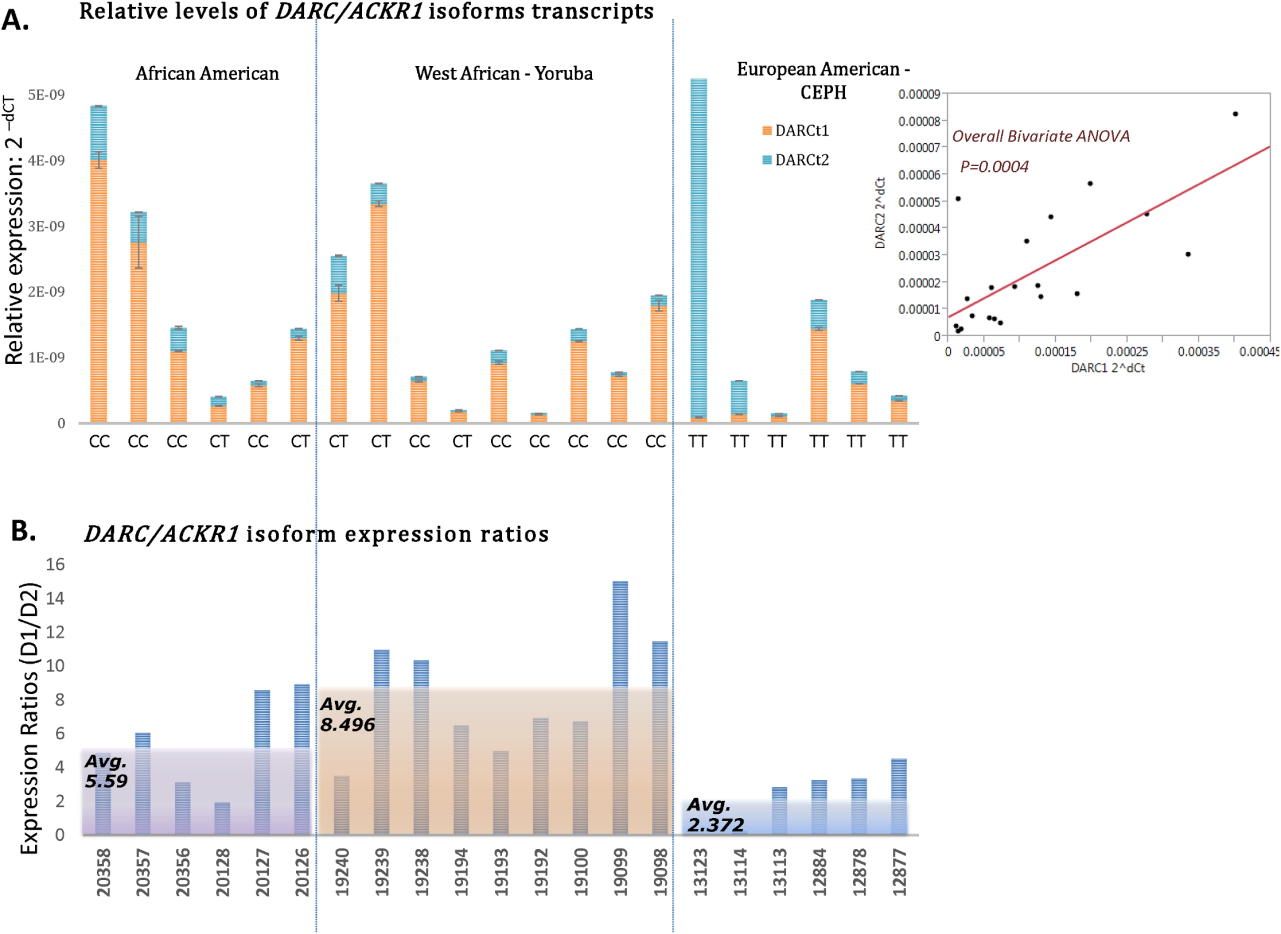
Differential expression of DARC isoforms in lymphoblasts derived from 1K Genomes populations. **(A)** Shows the relative expression levels of DARC 1 and DARC 2 transcripts among our HAPMAP cohort panel. The Fy- genotypes are indicated in the X axis of the top graph (A) and correlated HAPMAP cell line IDs are indicated in the X axis of the bottom graph (B). The “C” allele indicates the *Duffy Null* mutation. There is a clear trend of higher expression in the African and African Americans, with DARC 1 showing prominent expression. **(B)** Shows the relative ratios of DARC1/DARC2 isoforms in our cohort. The average ratio values for each ancestry category are shown as overlapping shaded box insets to display the values of each ancestry group. The highest ratio in the African category indicates the greatest difference between isoform expression values. C. ANOVA statistics indicate there are significant differences in DARC isoform transcripts across the cohort groups. The overall fitness across the entire group is mainly due to correlated expression of the isoforms for individuals with the C allele. There is clear trend of higher expression of the DARC 1 isoform in African and African American lineages, relative to European Americans. These data show a trend for lower expression in the European American (CEPH) categories and TT homozygotes for both DARC isoforms. These data indicate differences in isoform regulation, associated with the Fy- “*Duffy Null*” allele.

Using the well-defined genetic ancestry HAPMAP cohort, we investigated whether the *Fy*- genotype was associated with differential expression of *DARC* isoforms. We anticipated that the *Fy*- allele may facilitate removal of *DARC/ACKR1*expression from lymphocytes if the hematopoietic transcription factor (GATA-3), reported to be the regulator in erythrocytes [49], was also the regulator in lymphoblasts. Therefore, we decided to determine if this would also be true of lymphocyte lineages. However, we found the opposite to be true. Using RT-PCR we were able to detect mRNA expression of both *DARC*/ACKR1 isoforms, (DARC1/A and DARC2/B) in all individuals, including those with *Fy*- genotypes. However, the levels of DARC isoform transcripts were very dynamic among the cohort. Therefore, we quantified the differential levels of isoform-specific transcripts using qPCR (Fig 3A). An ANOVA across the entire group indicated the overall isoform transcript levels were significantly variant (p = 0.0007), suggesting the transcripts have distinct expression among our lymphoblast lines, derived from divergent ancestries (Fig 3A). The African and African American groups have a higher level of expression in these lines relative to CEPH-European Americans. The lower level of *DARC/ACKR1* isoform transcripts also correlates with the lower IHC scores in this population (Fig 2B). These findings illustrate the *Fy-* allele may be linked to altered expression of the gene in different cell types or tissues and not just removal of its expression in erythrocytes.

To visualize the relationship of *DARC/ACKR1* isoforms relative to each other within individuals, we calculated the relative expression ratios of [DARC1/DARC2] (Fig 3B). This perspective reveals a significant difference in isoform transcript levels among the ancestry groups. The West African group shows the highest average ratio, indicating the largest difference between isoform regulation. Additionally, the CEPH group shows the lowest average ratio, indicating that while the levels of *DARC/ACKR1* are lower in this population, the relative levels between isoforms is more correlated and may be suggestive of distinct promoter regulation differences. Accordingly, the African American ratios are averaged between the YRI and CEPH groups, similar to the genotype distributions.

### Differential *DARC* isoform expression is associated with genetic ancestry and *Fy-*genotypes

**Fig 4:**
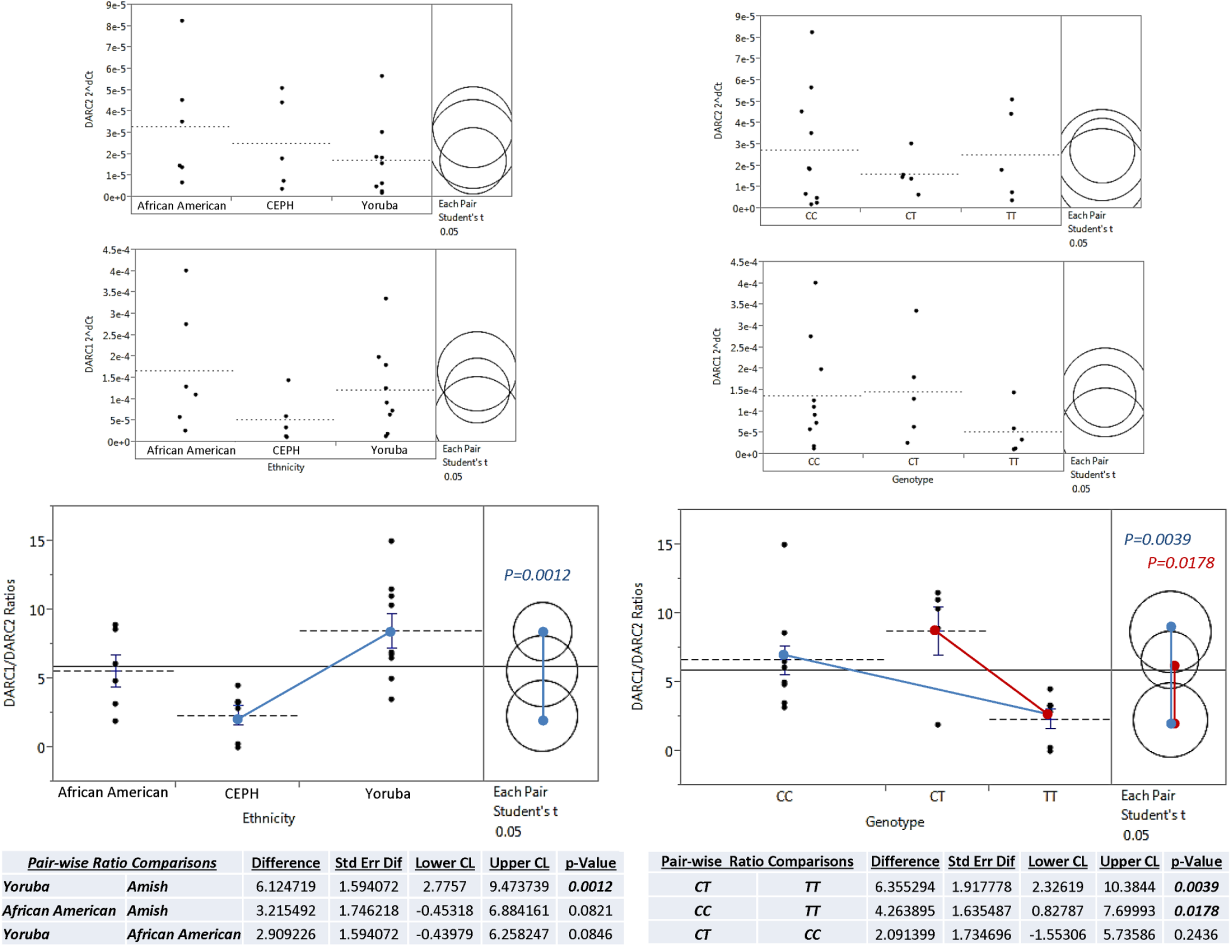
Statistical comparisons of relative levels of DARC isoform transcripts and ratios among ancestry and Fy- genotype groups. Statistical analyses across ancestry groups are on the left and genotype groups are on the right. ANOVA graphs indicate the individual group means (dotted blue line) of DARC isoform expression (top) and ratios (bottom) for stated categories. No statistically significant difference in individual transcript isoforms was identified across groups; however, significant differences were measured among ratios of DARC1/DARC2 isoforms. The means connection lines (solid blue and red) indicate the statistically significant comparisons. The significant p-values for pairwise T-test analyses of means is shown in the colors corresponding with each pair. The only statistically significant comparison across ancestry groups were between Amish and Yoruba groups. Across genotypes, there was a significant difference in DARC isoform ratios between TT genotype and both CT and CC genotypes. Significant differences between the isoform ratios indicate the isoforms are differentially regulated and the control of regulation is altered in isoform promoters with the C allele (Fy-).

To thoroughly interrogate the differential expression of *DARC/ACKR1* isoforms, we conducted a series of statistical tests to determine associations with either ancestry or the Fy- genotype. First, to determine if the specific isoform levels are associated with ethnicity, we conducted an ANOVA for each isoform across ethnicities. While we detected a trend for relatively lower and more tightly regulated levels in the EA group for both isoforms (Fig 4); however, this finding isn’t statistically significant (p = 0.482) possibly due to our small sample size. Similarly, when an ANOVA was conducted for each isoform across *Fy*- genotype groups, we found trends of expression levels with higher levels of both isoforms in individuals with the *Fy*- alleles.

Given that the ratio of DARC1/DARC2 revealed the relationship of relative isoform expression, we conducted pair-wise comparisons on these values to determine if relative levels of DARC1/DARC2 isoforms was associated with ancestry or Fy- genotypes. The results were significant. Across ancestry groups, the largest and only significant difference in relative isoform expression was between the CEPH and YRI categories (Pvalue = 0.0012). The African American group was an equal medium between the YRI and CEPH group and not significantly different from either. This observation also reflects the genotype distributions previously described. Across Fy- genotypes there was a significant difference between the categories with the “C” allele compared to the homozygous T allele. This finding indicates that the *Duffy Null Fy*- genotype results in significantly altered regulation and expression of the *DARC* isoforms and this is associated with African Ancestry.

## Conclusions

### Impact of altered *DARC* gene isoform levels among ancestry groups

IHC analysis indicates varying levels of *DARC/ACKR1* gene product among our cohort ancestry groups with the consistently the higher levels of in the African group lymphoblasts. This is in contrast to the *Duffy Null* phenotype status of these individuals, where of no *DARC/ACKR1* is expressed on erythrocytes. Intriguingly, the transcript variant reported to be impacted by the *Fy*- allele (*DARC/ACKR1-B*) is not the transcript affected in lymphoblast cells. Our results indicate that the levels of the *DARC/ACKR1-A*/Isoform A transcript show a significantly higher expression level among *Fy*- genotypes relative to DARC2 isoforms levels. The implications of this altered isoform regulation could have a huge impact on our understanding of *DARC/ACKR1* functionality. For instance, to date the repertoire of *Duffy* antigens includes only 2 distinct protein products, the result of the *Fy* ^*a/b*^ allele variation in the coding region. However, we have shown that the predominant transcript variant, in lymphoblasts, is the *DARC/ACKR1-A* isoform, which corresponds to the 338aa product variant. All studies to date have only addressed the 336aa product variant. This leaves a huge void of information concerning the distinct functions of the isoforms and especially in the context of the role DARC/ACKR1 plays in immunobiology; specifically, the impact of immunogenic potential for the 17 distinct amino acids between the isoforms, the potential of interactions between isoforms, the potential for isoform-specific affinity to certain chemokines as well as the tissue specificity or isoform spatial expression that relates to each of these characteristics. Summarily, we can conclude that each isoform expressed in an individual will also harbor the *Fy* ^*a*^ or *Fy* ^*b*^ allele, yielding 4 distinct *DARC/ACKR1* protein products for individuals who are heterozygous at the Fy^*a/b*^ locus. Of note, given our findings, we can confidently predict that certain individuals are likely expressing up to 4 different protein variants of *DARC/ACKR1* in circulating cells (Table 1) given the expression levels of transcripts (Fig 1) and levels of DARC/ACKR1 protein products (Fig 2). The combinations of these *Duffy* antigens could impact hemolytic interactions as well as regulation of chemokine levels in circulation. Indeed, perhaps the unexplained complexity with regard to alternate *Duffy* antigens (Fy5, Fy 6, etc.) could be due to the previously ignored isoform variants. In fact, *DARC/ACKR1* is a glycoprotein with unique prediction sites for extracellular, isoform-specific N-terminal glycosylation and therefore the potential for distinct isoform-specific interactions with chemokines is highly likely.

**Table 1:** Summary of DARC/ACKR1 genotyping for Duffy null and A/B alleles, DARC/ACKR1 protein isoform expression and predicted Duffy antigen phenotypes.

Ancestry	Cell Line	DARC isoform 1 mRNA	DARC isoform 2 mRNA	rs2814778 Fy^es-^ allele	rs12075 Fy^a/b^ allele	Expected Erythroid Phenotype	Anticipated Epithelial Phenotype	Potential # of antigens on RBC	Potential # of antigens on WBC
*Amish*	*GM12877*	*+*	*+*	*TT*	*AG*	*Fy (a+b+)*	*Fy (1a+1b+2a+2b+)*	*2–4*	*4*
	*GM12878*	*+*	*+*	*TT*	*AG*	*Fy (a+b+)*			
	*GM12884*	*+*	*+*	*TT*	*AG*	*Fy (a+b+)*			
	*GM13113*	*+*	*+*	*TT*	*AG*	*Fy (a+b+)*			
	*GM13114*	*+*	*+*	*TT*	*AG*	*Fy (a+b+)*			
	*GM13123*	*+*	*+*	*TT*	*AG*	*Fy (a+b+)*			
*West African—Yoruba*	GM19098	+	+	TC	AA	Fy (a-b+)	Fy (1a^***-***^ **,**1b^***+***^ **,**2a^***-***^ **,**2b^***+***^)	1–2	2
	GM19099	+	+	CC	AA	Fy (a-b-)	Fy (1a^***-***^ **,**1b^***+***^ **,**2a^***-***^ **,**2b^***+***^)	0	2
	***GM19100***	***+***	***+***	***CC***	***AG***	***Fy (a-b-)***	***Fy (1a+1b+2a+2b+)***	***0***	***4***
	GM19192	+	+	CC	AA	Fy (a-b-)	Fy (1a^***-***^ **,**1b^***+***^ **,**2a^***-***^ **,**2b^***+***^)	0	2
	GM19193	+	+	CC	AA	Fy (a-b-)	Fy (1a^***-***^ **,**1b^***+***^ **,**2a^***-***^ **,**2b^***+***^)	0	2
	GM19194	+	+	CC	AA	Fy (a-b-)	Fy (1a^***-***^ **,**1b^***+***^ **,**2a^***-***^ **,**2b^***+***^)	0	2
	GM19238	+	+	CC	AA	Fy (a-b-)	Fy (1a^***-***^ **,**1b^***+***^ **,**2a^***-***^ **,**2b^***+***^)	0	2
	GM19239	+	+	CC	AA	Fy (a-b-)	Fy (1a^***-***^ **,**1b^***+***^ **,**2a^***-***^ **,**2b^***+***^)	0	2
	GM19240	+	+	CC	AA	Fy (a-b-)	Fy (1a^***-***^ **,**1b^***+***^ **,**2a^***-***^ **,**2b^***+***^)	0	2
*African American*	***GM20126***	***+***	***+***	***TC***	***AG***	***Fy (a-b+)***	***Fy (1a*** ^***+/-*,**^ ***1b*** ^***+*,**^ ***2a*** ^***+/-***^ ***2b*** ^***+***^ ***)***	***1–2***	***2–4***
	GM20127	+	+	CC	AA	Fy (a-b-)	Fy (1a^***-***^ **,**1b^***+***^ **,**2a^***-***^ **,**2b^***+***^)	0	2
	***GM20128***	***+***	***+***	***TC***	***AG***	***Fy (a-b+)***	***Fy (1a*** ^***+/-***^ **,*1b*** ^***+***^ ***2a*** ^***+***^ ***/-2b*** ^***+***^ ***)***	***1–2***	***2–4***
	GM20356	+	+	TC	AA	Fy (a-b+)	Fy (1a^***-***^ **,**1b^***+***^ **,**2a^***-***^ **,**2b^***+***^)	1–2	2
	GM20357	+	+	CC	AA	Fy (a-b-)	Fy (1a^***-***^ **,**1b^***+***^ **,**2a^***-***^ **,**2b^***+***^)	0	2
	GM20358	+	+	CC	AG	Fy (a-b-)	Fy (1a^***-***^ **,**1b^***+***^ **,**2a^***-***^ **,**2b^***+***^)	0	4

We genotyped the Duffy null and Fy A/B alleles in our HapMap Cohort to validate the genotypes of the 1000 genomes populations of Amish, Yoruba and African Americans with expected results. The distribution of the genotypes for both the Fy- and Fy^a/b^ alleles show correlation with ancestry groups. We have detected the expression of DARC/ACKR1 isoforms in all lines and based on this have predicted the Duffy antigens anticipated to be expressed in these cells as well as other epithelial cells. Strikingly, we find that individuals who are erythrocyte-silent (es) null (alias-Duffy null) and express no Duffy antigens on red blood cells may express up to four different Duffy antigens on lymphoblasts (*italics*). Additionally, only two antigens are traditionally noted for individuals with Fy+ genotypes (Fy^a^ or Fy^b^); however, we have detected the expression of both isoform transcripts in these individuals which suggests there could be as many as 4 antigens expressed, even in erythrocytes ***(bold italics)***. Lastly, African Americans have a high frequency of compound heterozygosity, yielding a unique range of potential Duffy antigens on both the erythrocytes and lymphoblasts which may also translate to epithelial cells. Because the Duffy null and A/B alleles tend to be linked, and the Duffy positive allele tends to segregate with the Fy^b^ allele, this suggests most of the African American compound heterozygotes will express the B antigen on erythrocytes. In addition, because both transcript isoforms are expressed off the same gene (harboring the Fy^b^ allele) this will potentially result in expression of two separate antigens on erythrocytes and potentially up to 4 in lymphoblasts or other epithelial cells.

Of utmost importance, with regard to ancestry and *Fy*- individuals, we have now begun to uncover variation in *DARC/ACKR1* isoform levels in distinct tissues which may translate into variation of tissue-specific inflammatory responses, including those observed in the tumor microenvironment. With this study, we have begun to investigate distinct isoform-specific functions by characterizing these isoform differences among ancestry groups. More studies are now required to investigate how these isoforms function, which may completely transform our understanding of inflammatory actions within the vast array of chemokines, even with regard to known *DARC/ACKR1—*chemokine interactions among different classes of chemokines—none of which have been defined with respect to the isoforms. The physiologic consequences of differences in *DARC/ACKR1* isoform levels in *Fy*+ and *Fy*- individuals is completely unexplored and we hypothesize that distinct *DARC* isoform expression patterns will define a unique inflammatory status among *Fy*- individuals which may systemically influence disease susceptibility and clinical outcome, overall an array of diseases in which DARC has already been implicated to influence.

## Supporting Information

S1 Fig Current depiction of DARC spatial expression shows little or no expression in lymphocytes. Systemic expression levels of DARC in humans. Data from indicated microarray expression datasets were used to generate an eFP Browser image that depicts the unbiquitous expression of DARC/ACKR1 in the skeletal, digestive and immune systems. Adapted from the eFP by R.Patel. Images by E.T. Hamanishi. Data from GSE1133, E-GEOD-7307, GSE3526, GSE2361, GSE19650, E-GEOD-6257. Data normalized by MAS 5.0 method TGT value 100
(TIFF)

S1 Table 1000 Genomes Population Abbreviations. List of abbreviations for the 1,000 Genomes populations mentioned in Fig 1.
(PNG)
