# Supplementary figures and images for: Distinct Transcript Isoforms of the Atypical Chemokine Receptor 1 (ACKR1) / Duffy Antigen Receptor for Chemokines (DARC) Gene Are Expressed in Lymphoblasts and Altered Isoform Levels Are Associated with Genetic Ancestry and the Duffy-Null Allele
Source: PLoS One. 2015 Oct 16;10(10):e0140098. doi: 10.1371/journal.pone.0140098 (PMC4608815; doi:10.1371/journal.pone.0140098)

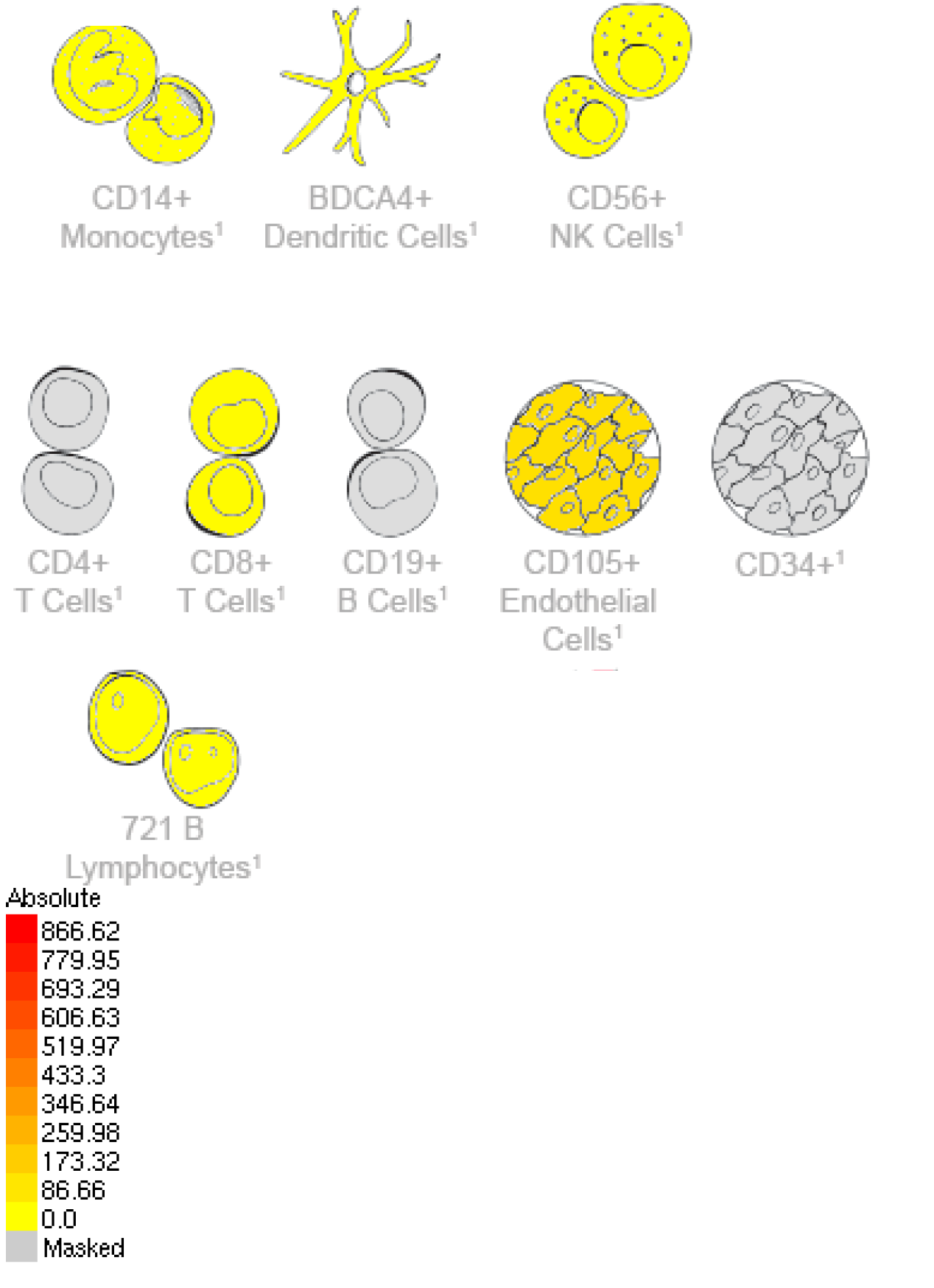

Supplement: S1 Fig — Systemic expression levels of DARC in humans. Data from indicated microarray expression datasets were used to generate an eFP Browser image that depicts the unbiquitous expression of DARC/ACKR1 in the skeletal, digestive and immune systems. Adapted from the eFP by R.Patel. Images by E.T. Hamanishi. Data from GSE1133, E-GEOD-7307, GSE3526, GSE2361, GSE19650, E-GEOD-6257. Data normalized by MAS 5.0 method TGT value 100 (TIFF) [file pone.0140098.s001.tiff]

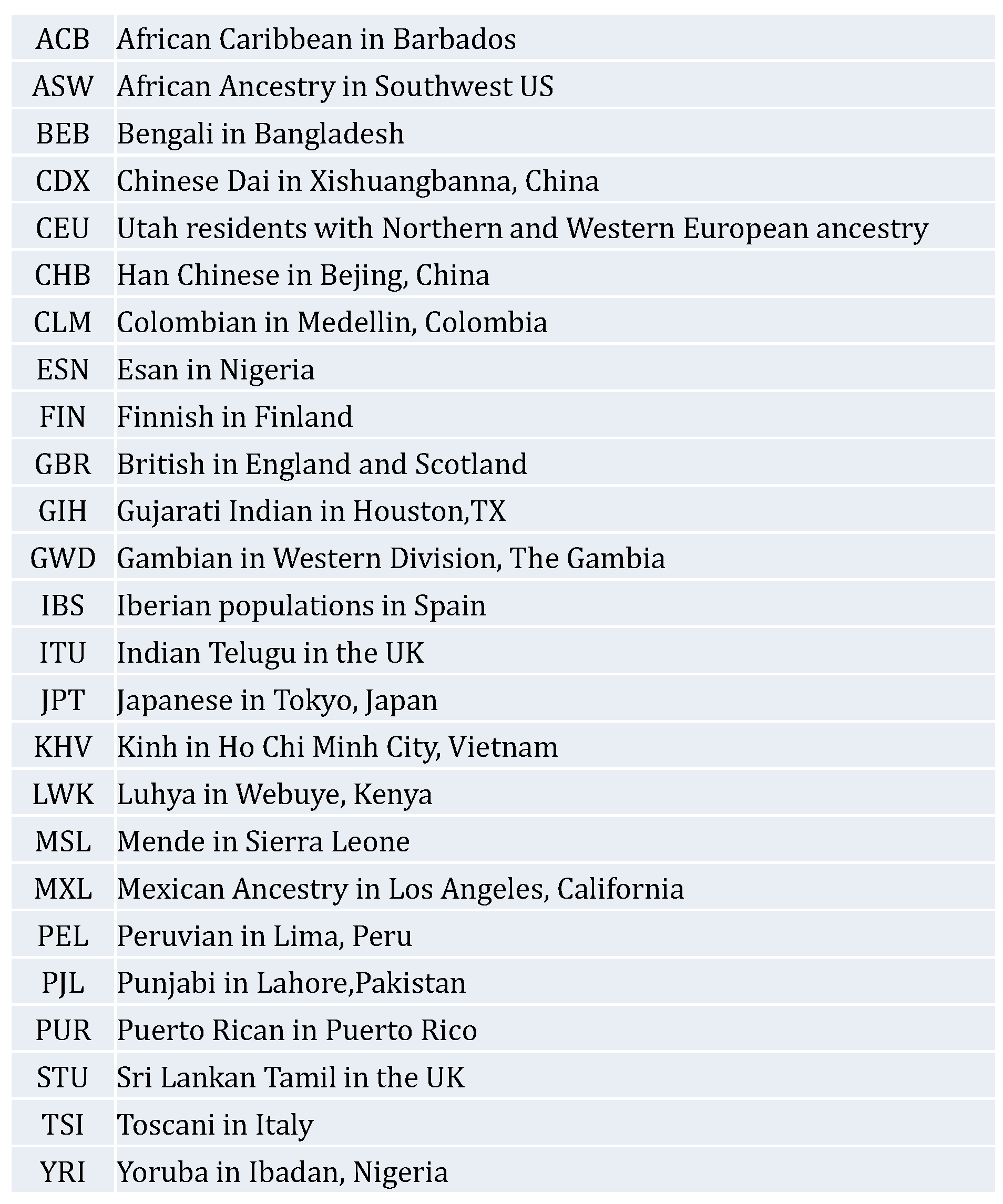

Supplement: S1 Table — List of abbreviations for the 1,000 Genomes populations mentioned in Fig 1. (PNG) [file pone.0140098.s002.png]
